# Psychometric characteristics of the Iranian Caregiver Burden Inventory (CBI) in caregivers of elderly patients with Alzheimer

**DOI:** 10.1186/s12955-020-01509-7

**Published:** 2020-07-29

**Authors:** Akram Shafiezadeh, Majideh Heravi-Karimooi, Amin Mirzaee, Nahid Rejeh, Hamid Sharif Nia, Razieh Bandari, Ali Montazeri

**Affiliations:** 1 College of Nursing & Midwifery, Shahed University, Tehran, Iran; 2 Elderly Care Research Centre, College of Nursing & Midwifery, Shahed University, Tehran, Iran; 3 Behavioral Sciences Research Center, Life Style Institute, Baqiyatallah University of Medical Sciences, Tehran, Iran; 4 School of Nursing & Midwifery Amol, Mazandaran University of Medical sciences, Sari, Iran; 5 Social Determinants of Health Research Center, Semnan University of Medical Sciences, Semnan, Iran; 6 Health Metrics Research Centre, Iranian Institute for Health Sciences Research, ACECR, Tehran, Iran; 7 Faculty of Humanity Sciences, University of Science & Culture, ACECR, Tehran, Iran

**Keywords:** Caregiver burden, Psychometrics, Alzheimer’s elderly, Caregiver burden inventory

## Abstract

**Background:**

It is essential to better understand the caregiving burden and its determinants to support caregivers. The aim of this study was to test the validity and reliability (internal consistency) of the caregiver burden inventory (CBI) in Iranian caregivers of elderly patients with Alzheimer.

**Methods:**

A cross sectional study was conducted to translate and validate the CBI in Iran. The structural validity of the scale was evaluated by exploratory factor analysis. The concurrent validity was tested correlating the CBI scores with the Beck Anxiety Inventory (BAI) and the Beck Depression Inventory (BDI) scores. The internal consistency reliability was assessed using the Cronbach’s alpha coefficient.

**Results:**

In total, we enrolled 315 caregivers of elderly patients with Alzheimer disease (79% women, mean age 56.5 ± 13.9). The structural validity of the CBI as assessed by exploratory factor analysis indicated three factors (emotional/psychological, time-dependence and physical burden) that jointly explained 45.21% of the total variance observed. The Pearson correlation coefficient for the relationship between the scores obtained on the Caregiver Burden Inventory and the BAI and the BDI were 0.69 and 0.49 respectively, confirming the concurrent validity. The Cronbach’s alpha for the CBI was 0.91 ranging from 0.78 to 0.92 for its subscales.

**Conclusion:**

Used in clinical practice and research, the CBI was verified to be a proper multidimensional instrument for evaluating the burden in caregivers of elderly patients with Alzheimer disease.

## Background

The care burden is defined as the perceived negative impacts of caring for a family member [1]. The conceptualization of care burden has evolved over time. It was first considered as a one-dimensional construct and was originally defined as a family cost [2]. It was then considered as a two-dimensional concept including the objective (activities and needs for care) and subjective burden of care (attitudes and emotional reactions to care) [3]. The care burden is currently considered as a multidimensional construct encompassing social, emotional, psychological, physical, and economic consequences [4, 5]. This conceptualization of care burden is derived from the caregiver population and includes direct and indirect stressors.

The emotional, physical, and social well-being of caregivers is often neglected, making the caregiver as a forgotten patient [6]. Many studies have emphasized that the burden of care has a negative impact on the patients’ health, the quality of care provided by the caregivers, and the caregivers’ well-being [7]. Indeed, those caregivers who experience high levels of care burden are at greater risk for psychological distress, anxiety, depression and lower quality of life [8–10].

Studies showed that support provided by caregivers for the patients with cognitive impairments, such as Alzheimer disease could improve outcomes [11]. However, patients’ need for care increases as the disease progresses, so that in the late stages of the disease, patients usually need 24-h care. Thus, the primary burden of care for patients with Alzheimer disease fell on the patients’ family shoulders, which affects many aspects of their life [12]. As a result, caregivers might neglect or mistreat their patients [13]. Therefore, the evaluation and diagnosis of care burden in caregivers of patients with Alzheimer disease is crucial.

At present among existing instruments that evaluates care burden, the Caregiver Burden Inventory (CBI) seems more popular [14]. The instrument has a multidimensional approach to the concept of caregiver burden and has been widely used in different research settings. It evaluates various aspects of care burden and can help to identify the specific needs of caregivers [15]. The CBI is validated in Italy [9] and China [16] for caregivers of the elderly patients with Alzheimer disease, and in Brazil [17] and Spain [18] for the caregivers of elderly patients with other various diseases. These studies have confirmed that the CBI is a valid and reliable tool for measuring caregivers’ burden of elderly patients in different cultures and contexts. Thus since the instrument was not available in Persian, the purpose of this study was to translate and assess the psychometric properties of the CBI among caregivers of elderly patients with Alzheimer disease in Iran.

## Methods

### Participants and study setting

A methodological study was conducted at the Alzheimer Disease Association of Iran in Tehran. As such a sample of caregivers of elderly patients with Alzheimer were entered into the study. The required sample was estimated based on availability and recommendations for performing exploratory factor analysis. Although there is no optimal suggestion for the appropriate sample size when conducting a factor analysis [19], some investigators recommended that for factor analysis, a sample of 100 participants is inadequate, 200 as relatively good, 300 as good, 500 as very good, and 1000 as high [20]. However, we included 315 caregivers of elderly patients with Alzheimer disease on a voluntary basis (i.e., convenience sampling). In order to minimize the drop out, data collection strategies were used for cross-sectional studies such as engaging study participants, and talking to participants with kindness and affection [21]. In order to be included in the study, the participants should have: taken care of the elderly patients with Alzheimer disease, and signed the informed consent form. The study exclusion criteria were: having any communication problems (e.g. inability to read or write), any conditions that may hinder continued caregivers’ participation in the study (e.g. severe cognitive problems, severe visual and hearing impairment), any medical conditions or psychological treatment during 2 months prior to the study.

### Data collection and procedures

When caregivers were attending to the Alzheimer Disease Association of Iran for medical care of their patients we introduced the study and they were asked to participate in the study and respond to the questionnaires if they wish. The informed consent was obtained after informing the participants about the purpose of the study and their right to withdraw it at any time. Answering the study questionnaires took approximately 10 min.

### Instrument: The Caregiver Burden Inventory (CBI)

The Caregiver Burden Inventory (CBI) includes 24 items and 5 dimensions: Time-dependence, developmental, physical, emotional, and social burden. There are 5 items for each dimension, except for the physical dimension, which has four items. Each item signifies a score between zero (not descriptive) and four (highly descriptive), with a higher score indicating greater care burden. Therefore the total score for time-dependence, developmental, emotional, and social burden range from 0 to 20 except for physical burden where scores range from 0 to 16. However, as suggested the score on physical burden also could be multiplied by 1.25 to obtain an equivalent score out of 20 [14].

### Translation procedure

The World Health Organization protocol of forward-backward translation technique was used to translate the scale from English into Persian [22]. The English version of the CBI was first translated into Persian by two researchers who were fluent in English. Then, two independent translators who did not previously see the English version of the CBI, back translated the questionnaire into English. The translators discussed the disagreements in translations with the original version and a final consensus was reached. Lastly the research team examined the semantic, empirical, terminological and conceptual balance between the original CBI version and the Iranian version. The final version of the CBI was given to 10 caregivers who did not participate in this study to ensure that caregivers could understand the CBI.

### Additional measures

#### Demographic information of caregivers and elderly patients with Alzheimer disease

Self-reported demographic information such as age, gender, education level, caregiver’s place of residence (with or without the patient, duration of care for the patient) were collected from the elderly with Alzheimer’s disease and caregivers.

#### Beck Anxiety Inventory (BAI)

The Beck Anxiety Inventory contains 21 items and measures the levels of anxiety. Each item shows one of the symptoms of anxiety by those who experienced anxiety or were in anxious situations [23]. The psychometric properties of Beck Anxiety Inventory in Iran were studied and the Cronbach’s alpha was 0.93 [24].

#### Beck Depression Inventory (BDI)

The Beck Depression Inventory consists of 21 items tapping into 3 cognitive, motivational, and psychological dimensions. Items are rated on a Likert scale ranging from 0 to 3 [25]. The psychometric properties of the Iranian version of the questionnaire are well documented and the Cronbach’s alpha for the inventory was reported to be 0.93 [26].

### Structural validity

To evaluate the structural validity, the maximum likelihood factor structure of the Persian version of the CBI was investigated by exploratory factor analysis using the varimax rotation. All analyses were performed using the SPSS 22 (SPSS Inc., Chicago, IL, USA). The Kaiser-Meyer-Olkin (KMO) test and Bartlett’s test of sphericity were used to check the appropriateness of the study sample and the factor analysis model. The number of latent factors was estimated via parallel analysis. As recommended we considered the items with absolute loading values of 0.4 or greater since the sample size was more than 300 individuals [27].

### Reliability assessment

The Cronbach’s alpha was estimated in order to assess reliability internal consistency of the CBI. The alpha value of 0.7 or greater was considered satisfactory [28].

### Missing data

The missing data were evaluated via multiple imputations, and replaced by the average responses [29].

## Results

### The characteristics of study sample

The mean age of caregivers was 56.5 (SD = 13.9) years and it was 77.9 (SD = 10.2) for patients. Most caregivers were female (78.7%) and living with patients (58.4%). One hundred and seventy five patients (55.5%) were women and the prevalence of moderate to severe dementia among patients was 19.0 and 57.8%, respectively. Table 1 presents the characteristics of caregivers and patients.

**Table 1:** The characteristics of caregivers and patients with Alzheimer disease (*n* = 315)

	Caregivers	Patients
**Gender** (No.%)		
Female	248 (78.7)	175 (55.5)
Male	67 (21.3)	140 (44.5)
**Age** (Mean, SD)	56.5 ± 13.9	77.9 ± 10.2
**Education** (No., %)		
Illiterate/primary	55 (17.5)	196 (62.2)
Secondary/higher	260 (82.5)	119 (37.8)
**Marital status**		
Married	240 (76.2)	85 (27.0)
Unmarried/widowed	75 (23.8)	230 (73.0)
**Employment status**		
Currently employed	94 (29.8)	5 (1.6)
Unemployed	221 (70.2)	310 (98.4)
**Relationship with patient**		
Spouse	85 (26.9)	NA
The child of patient	180 (57.1)	NA
Other family or nurse	50 (15.9)	NA
**Living condition** (No., %)		NA
Living with patient	184 (58.4)	NA
Not living with patient	131 (41.6)	NA
**Hours of care giving per day** (Mean, SD)	7.6 ± 7.2	–
**Patients’ dementia severity** (No. %)		
Severe	NA	60 (19.0)
Moderate	NA	182 (57.8)
Mild	NA	73 (23.2)
**Duration of dementia** (months/Mean, SD)	NA	36.8 (41.8)

*NA* Not applicable

### Structural validity

An exploratory factor analysis with varimax rotation was used to assess the structural validity of the questionnaire. The Kaiser-Meyer-Olkin adequacy was 0.93 and Bartlett’s test of sphericity was significant (*p* <  0.001) indicating the sample adequacy. After rotation, 24 items were loaded on four factors that jointly accounting for 52.8% of variance. However, after removing 5 items due to low loadings (< 0.4) (item 16, 15, 20.24,11), the remaining 19 items were reanalyzed and loaded on three distinct factors that accounted for 49.79% of variance observed. In fact items belonging to developmental, and emotional burden in addition to item 17, and 19 from social burden were clustered to form one cluster, which we named this new factor as emotional/psychological burden. The other two factors included item 1, 2, 3, 4, and 5 (time-dependence burden), and item 12, 13, and 14 (physical burden), respectively. The results are shown in Table 2.

**Table 2:** The results obtained from factor analysis for the Persian version of CBI in caregivers of elderly patients with Alzheimer disease

Items	F1	F2	F3
7. I wish I could escape from this situation	**0.876**	0.160	−0.053
8. My social life has suffered	**0.863**	0.101	−0.055
18. I don’t do as good a job at work as I used to	**0.739**	−0.163	0.357
9. I feel emotionally drained, due to caring for my care receiver	**0.704**	0.088	.076
19. I feel resentful of other relatives who could but do not help	**0.683**	0.051	0.241
6. I feel that I’m missing out on life	**0.674**	−0.150	0.120
22. I resent my care receiver	**0.660**	−0.017	0.059
17. I’ve had problems with my marriage	**0.560**	−0.053	0.095
10. I expected that things would be different at this point in my life	**0.538**	0.189	0.079
21.I feel ashamed on my care receiver	**0.516**	0.004	0.012
23. I feel uncomfortable when I have friends over	**0.497**	0.077	0.095
4. I have to help my care receiver with many basic functions	0.339	**0.868**	0.017
1. My care receiver needs my help to perform many daily tasks	0.218	**0.835**	0.002
5. I don’t have a minute’s break from my caregiving chores	0.258	**0.810**	−0.071
2. My care receiver is dependent on me	0.084	**0.713**	0.018
3. I have to watch my care receiver constantly	0.030	**0.505**	−0.004
13. Caregiving has made me physically ill	0.094	0.160	**0.993**
12. My health has suffered	−0.087	−0.047	**0.742**
14. I’m physically tired	−0.021	0.128	**0.615**
24. I feel angry about my reactions toward my care receiver	0.382	0.225	0.017
20.I feel embarrassed by my care receiver behavior	0.376	0.103	0.086
15. I don’t get along with other family members as well as I used to	0.371	0.077	0.095
11. I’m not getting enough sleep	0.360	0.199	0.002
16. My caregiving efforts aren’t appreciated by others in my family	0.354	0.271	0.123
*Eigenvalue*	9.79	2.46	1.23
*% of variance*	38.37	8.40	2.96
*Cronbach’s alpha*	0.90	0.78	0.92

*F1* emotional/psychological burden, *F2* time-dependence burden, *F3* physical burden

### Concurrent validity

The concurrent validity of the CBI was assessed using the correlation between the CBI scores and the BAI and the BDI scores. There were positive and significant correlations between these measures. As shown in Tables 3 and 4 the overall correlation between the Caregiver Burden Inventory and the BAI and the BDI were 0.69 and 0.49, respectively.

**Table 3:** Correlation between the BAI and the CBI

	BAI	Emotional/psychological burden	Time-dependence burden	Physical burden
**BAI**	1			
**Emotional/psychological burden**	0.65^**^	1		
**Time-dependence burden**	0.30^*^	0.38^*^	1	
**Physical burden**	0.64^**^	0.80^**^	0.37^*^	1
**CBI (total score)**	0.69^**^	0.95^**^	0.64^*^	0.85^**^

*BAI* Beck Anxiety Inventory, *CBI* Caregiver Burden Inventory

* *P* <  0.05, ** *P* <  0.001

**Table 4:** Correlation between the BDI and the CBI

	BDI	Emotional/psychological burden	Time-dependence burden	Physical burden
**BDI**	1			
**Emotional/psychological burden**	0.654^**^	1		
**Time-dependence burden**	0.302*	0.38^*^	1	
**Physical burden**	0.649^*^	0.80^**^	0.37^*^	1
**CBI (total score)**	0.49^**^	0.95^**^	0.64^**^	0.85^**^

*BDI* Beck Depression Inventory, *CBI* Caregiver Burden Inventory

* *P* <  0.05, **P < 0.001

### Reliability

As reported in Table 2, the Cronbach’s alpha was 0.90, 0.78, and 0.92 for the emotional/psychological, Time-dependence, and physical burden subscales, respectively. The alpha coefficient for the scale as a whole was 0.91.

### The CBI scores among sub-groups of the study sample

A bivariate analysis between independent variables (age, gender, educational level, employment status, relationship with patient, living condition, marital status, patients’ dementia severity) and the global burden and the three factors are shown in Table 5. As shown there were significant differences between total scores of caregivers’ burden and age, gender, relationship with patient, and patients’ dementia severity.

**Table 5:** Total and related CBI sub-dimension scores according to the caregivers’ characteristics

	F1	F2	F3	Total CBI	P*
	Mean (SD)	Mean (SD)	Mean (SD)	Mean (SD)	
**Age groups**					< 0.0001
20–40	12.62 (10.3)	12.98 (5.5)	3.95 (4.3)	29.56 (16.6)	
41–60	12.55 (9.4)	14.06 (6.6)	5.12 (4.6)	31.74 (17.0)	
61–80	18.47 (11.4)	14.42 (5.2)	7.39 (5.0)	40.28 (18.2)	
**Gender**					0.001
Male	9.91 (8.5)	12.97 (5.2)	3.43 (3.9)	26.31 (14.2)	
Female	15.34 (10.7)	14.09 (6.1)	5.97 (4.9)	35.37 (18.1)	
**Educational level**					0.108
Illiterate	16.34 (11.0)	14.15 (10.5)	6.29 (5.1)	38.04 (17.4)	
Primary	14.19 (9.6)	15.09 (5.06)	6.07 (4.8)	35.52 (15.7)	
Secondary	13.84 (11.4)	15.25 (4.8)	5.25 (4.9)	32.58 (18.9)	
Higher	13.40 (9.7)	13.74 (7.1)	4.93 (4.6)	31.34 (17.0)	
**Employment status**					0.960
Currently employed	14.16 (10.4)	13.87 (6.1)	5.42 (4.8)	33.36 (17.6)	
Unemployed	14.08 (11.2)	13.82 (5.3)	5.31 (4.8)	33.51 (18.4)	
**Relationship with patient**					< 0.0001
Spouse	19.17 (11.5)	15.28 (6.9)	7.55 (4.9)	42.03 (18.2)	
The child of patient	12.57 (9.3)	13.63 (5.3)	4.88 (4.6)	31.08 (16.0)	
Other family or nurse	11.32 (10.1)	12.32 (6.3)	3.65 (4.5)	27.29 (17.6)	
**Living condition**					0.08
Living with patient	16.39 (11.3)	14.85 (6.1)	6.10 (5.0)	37.30 (18.1)	
Not living with patient	11.01 (8.4)	12.48 (5.5)	4.43 (4.4)	27.90 (15.5)	
**Marital status**					0.542
Married	13.99 (10.5)	13.83 (5.4)	5.51 (4.9)	33.29 (17.5)	
Unmarried/widowed	14.68 (10.5)	13.96 (7.5)	5.06 (4.7)	33.70 (18.3)	
**Patients’ dementia severity**					0.001
Severe	16.06 (9.9)	15.53 (7.7)	6.27 (4.7)	6.27 (4.7)	
Moderate	14.81 (10.6)	14.37 (4.8)	5.73 (4.9)	5.73 (4.9)	
Mild	10.94 (10.1)	11.14 (6.2)	3.87 (4.6)	3.87 (4.6)	

*F1* emotional/psychological burden, *F2* time-dependence burden, *F3* physical burden

*Derived from t-test or one-way analysis of variance (ANOVA) comparing the total scores among different subgroups of the study sample

## Discussion

The results of the present study demonstrated that the CBI included three distinct and stable factors namely emotional/psychological, time-dependence, and physical burden. These factors jointly explained 45.21% of the variance observed. However, this was not similar to the original work by the Novak et al. where they extracted five factors for the questionnaire (time-dependence, developmental, physical, emotional, and social burden) [14]. Marvardi et al. in assessing the psychometric properties of the CBI in Alzheimer’s elderly caregivers in Italy showed that CBI consisted of four factors [9]. Molde et al. pointed out that different factor structures observed in several studies might be due to different reasons including cultural issues, linguistic aspects, and sample characteristics [30].

The present study validated the caregiver burden inventory in Iran for the first time. The exploratory factor analysis was used to evaluate the structural validity of the questionnaire. The first factor identified in our study was emotional/psychological burden. There is evidence that family members of patients with dementia and Alzheimer disease are usually forced to perform informal care alone without any training, orientation, or support from health professionals. It is argued that such conditions significantly increase their subjective and mental burdens and could lead to psychological problems such as depression and anxiety symptoms and impaired quality of life [31].

The second factor of the CBI scale found in this study was time-dependence burden. Time-dependence burden is related to the severity of the patient’s illness and the extent of caregiver involvement in patient care. It is strongly associated with those issues pertaining to how much attention the caregiver pays to the care recipient, particularly in terms of patient’s functioning and the number of care giving tasks that are being performed. So the high severity of illness and prolonged patient care increases the time-dependence burden [3].

The third factor identified in the present study was physical burden. This factor referrers to physical burden and somatic disorders [9]. The physical burden is significantly relating to the patient’s perceived health and duration of illness. Those caregivers who are less affluent and have been sick for more days’ bear more physical burden [31]. In line with this study, this factor appeared in several other studies as an important aspect of the CBI [9, 16–18].

The study findings revealed a significant moderate to good levels of correlation between the CBI and the BAI and the BDI lending support to the acceptable concurrent validity of the CBI. However, one should note that the coefficients for such correlations should be interpreted in the light of recommended values for these observations where values of *r* ≥ 0.81–1.0 are considered as excellent, 0.61–0.80 very good, 0.41–0.60 good, 0.21–0.40 fair, and 0–0.20 poor [28]. Similarly, previous studies have confirmed the concurrent validity of the CBI in other populations [9, 16, 18].

The internal consistency of the CBI as assessed by estimating the Cronbach’s alpha was acceptable (alpha value of 0.91). The high level of Cronbach’s alpha signifies the internal consistency of the scale and the correlation between the items. Novak et al. found that the Cronbach’s alpha coefficient for the CBI was 0.84 [14]. The reliability of this scale has been assessed by Cronbach’s alpha in several other studies. For instance, the internal consistency (Cronbach’s alpha) of the CBI was 0.89 in the study conducted by Vázquez et al. in Spain [18].

### Limitations

Although this study provided some useful results for future studies, it also had a number of limitations. The sample was restricted to a group of caregivers attending to the Alzheimer Disease Association of Iran in the Tehran. Thus, non-random sampling might limit the findings. In addition we used a number of limited psychometric evaluations. Perhaps there is need to conduct studies to assess other psychometric issues and examine the factor structure of the questionnaire once more.

### Implications for practice

Psychologists, nurses, and other specialists in medical and research centers can use the Persian version of the CBI to timely assess and diagnose caregivers’ burden. Timely diagnosis can prevent many psychological and physical problems in caregivers and even might improve their quality of life.

## Conclusion

The findings from this study confirmed the acceptable psychometric properties, as well as the factor structure of the CBI in an Iranian sample. Given these findings, the scale can be used as a valid, and reliable instrument to assess the burden experiencing by caregivers of elderly patients with Alzheimer disease.

## Data Availability

The datasets are available from the corresponding authors on request.

## Abbreviations

EFA: Exploratory Factor Analysis
KMO: Kaiser–Meyer–Olkin
CBI: Caregiver Burden Inventory;
BAI: Beck Anxiety Inventory;
BDI: Beck Depression Inventory
ICC: Intraclass Correlation Coefficients
