# Correction to: Psychometric characteristics of the Iranian Caregiver Burden Inventory (CBI) in caregivers of elderly patients with Alzheimer

**DOI:** 10.1186/s12955-021-01848-z

**Published:** 2021-09-06

**Authors:** Akram Shafiezadeh, Majideh Heravi-Karimooi, Amin Mirzaee, Nahid Rejeh, Hamid Sharif Nia, Ali Montazeri

**Affiliations:** 1 College of Nursing & Midwifery, Shahed University, Tehran, Iran; 2 Elderly Care Research Centre, College of Nursing & Midwifery, Shahed University, Tehran, Iran; 3 Behavioral Sciences Research Center, Life Style Institute, Baqiyatallah University of Medical Sciences, Tehran, Iran; 4 School of Nursing & Midwifery Amol, Mazandaran University of Medical Sciences, Sari, Iran; 5 Health Metrics Research Centre, Iranian Institute for Health Sciences Research, ACECR, Tehran, Iran; 6 Faculty of Humanity Sciences, University of Science & Culture, ACECR, Tehran, Iran

## Correction to: Health and Quality of Life Outcomes (2020) 18:255 10.1186/s12955-020-01509-7

The authorship of this article [1] has been corrected. Razieh Bandari contributed to the statistical analysis during the revision of the article and was added as an author without consent from all authors. The authors and Razieh Bandari have requested to remove her as an author and to update the authorship accordingly. Razieh Bandari’s contributions are acknowledged in the updated Acknowledgements section.
